# Impact of smoking on the incidence and post-operative complications of total knee arthroplasty: A systematic review and meta-analysis of cohort studies

**DOI:** 10.17305/bjbms.2021.6538

**Published:** 2021-12-20

**Authors:** Yuqi He, Mohamed Omar, Xiaoyuan Feng, Claudia Neunaber, Michael Jagodzinski

**Affiliations:** 1 Department of Trauma, Hannover Medical School, Hannover, Germany; 2 Department of Pain, Chengdu Second People’s Hospital, Chengdu, China; 3 Department of Orthopaedic Trauma, Agaplesion Ev. Klinikum Schaumburg, Obernkirchen, Germany

**Keywords:** Total knee arthroplasty, smoking, incidence, complications

## Abstract

Osteoarthritis and rheumatoid arthritis are the most ubiquitous joint disorders which cause tremendous loss of life quality and impose an economic burden on society. At present, the treatment options for these two diseases comprise non-operative and surgical treatments, among those total knee arthroplasty (TKA). Various studies have recognized smoking as a significant risk factor for post-operative complications. Therefore, the purpose of this study was to examine the impact of smoking on the incidence and post-operative complications after a TKA by a systematic review and meta-analysis. The research was performed using PUBMED, Cochrane Library and EMBASE, extracting data from thirteen suitable studies and incorporating 2,109,482 patients. Cohort studies evaluating the impact of smoking on TKA with sufficient data were included for the study, and cohort studies without a proper control group and complete data were excluded from the analysis. A fixed-effects or random-effects model was used to measure the pooled risk ratio or hazard ratio with 95% confidence interval (CI). Compared to non-smokers, smokers had a significantly lower incidence of TKA (*p* < 0.01). However, smokers had a higher incidence of total complications (*p* = 0.01), surgical complications (*p* < 0.01), pneumonia (*p* < 0.01), and revision surgery (*p* = 0.01). No significant difference in the risk of blood transfusion (*p* = 0.42), deep vein thrombosis (*p* = 0.31), pulmonary embolism (*p* = 0.34), urinary tract infection (*p* = 0.46), or mortality (*p* = 0.39) was found between smokers and non-smokers. In conclusion, the study indicated that tobacco has two diametrically opposite effects on TKA patients: (1) Tobacco increases the incidence of surgical complications, pneumonia and revision after TKA; (2) It decreases the overall risk of being a candidate for TKA.

## INTRODUCTION

Osteoarthritis (OA), which affects an estimated 240 million individuals globally with 8.6/1000 new cases per year [1], and rheumatoid arthritis (RA), which affects 0.5-1.0% of adults with 5-50/100,000 new cases per year [2], are the most ubiquitous joint disorders. They cause tremendous loss of life quality to patients and impose a tremendous economic burden on society. At present, the treatment options for these two diseases comprise non-operative and surgical treatments, among those total knee arthroplasty (TKA). When patients are diagnosed with OA or RA, the orthopedic surgeon is required to enhance a non-operative protocol, including regular medication [3]. Some patients can monitor their disease well with medication for the rest of their lives without needing surgical treatment, whereas some fail to respond to conservative means and finally need a TKA. Complications associated with surgery are wound infection, revision surgery, deep vein thrombosis and pulmonary embolism, leading to a mortality rate of about 0.5%, while 77% of the patients are satisfied with the result [4]. Various studies have recently shown that patients’ lifestyles can influence the outcomes of orthopedic disorders; smoking, for example, is widely recognized as a significant risk factor for post-operative complications [5]. After querying the American College of Surgeons National Surgical Quality Improvement Program database, Duchman et al. found that smokers had an increased risk of total complications than non-smokers following TKA [6]. Smoking patients need more narcotic and benzodiazepine prescriptions to achieve pain relief after TKA [7]. There was a 3-fold substantial increase in the 1-year risk of mortality among RA patients who were practicing tobacco smoking at the time of elective TKA surgery [8]. These studies seem to suggest that smoking harms OA or RA patients and TKA surgery. However, nowadays, some studies show different opinions on the relationship between smoking and TKA in OA and RA patients, suggesting that smoking can decrease the progression of OA, and also lower the risk of TKA in OA or RA patients [9,10]. This challenges the traditional view of the relationship between smoking and TKA. In 2013, Pearce et al. conducted a meta-analysis to find that smoking has no protective effect on OA progression [11], but several subsequent clinical studies have suggested that smoking is a protective factor for OA or TKA [12-15]. Meanwhile, some other studies focused on complications after TKA, but the outcome is not comprehensive enough. For example, only post-operative infections were compared, and revision rate, mortality, and internal medicine complications were not considered [16,17].

Hence, it is worthwhile to re-conduct a comprehensive meta-analysis to assess the overall effect of smoking on TKA in OA and RA patients, including the risk before surgery and to re-analyze the impact of smoking on complications after surgery. Due to investigate the real impact of smoking on TKA, we systematically reviewed all the relevant research and performed this meta-analysis from cohort studies.

## MATERIALS AND METHODS

A systematic literature review and a meta-analysis were performed to explore the impact of smoking on TKA, including the incidence of TKA following a diagnosis of OA or RA and the complications of TKA. This analysis was conducted according to preferred reporting items for systematic reviews and meta-analyses guidelines and was registered in the International Prospective Register of Systematic Reviews (PROSPERO, https://www.crd.york.ac.uk/PROSPERO, Identifier: CRD42021235436) [18].

### Literature search

A comprehensive search was conducted across multi-databases (PubMed, Cochrane and EMBASE) from the date of database inception up till February 5, 2021. The keywords and Boolean operator terms utilized for the search were summarized briefly as follows: ([“TKA” OR ‘Total knee replacement”] AND [“Smoke*” OR “Tobacco*” OR “Nicoti*”]) (see the detailed search strategy in Supplement Table 1). Identified studies and their related references have been checked by the selection criteria for inclusion.

### Study selection criteria

Two independent authors carried out a literature search, and a third author was consulted in the event of any differences of opinion. Studies included have been objectively analyzed using a predefined data extraction form. Inclusion criteria for the meta-analysis were as follows: (1) cohort study design including retrospective and prospective studies, (2) evaluated the impact of smoking on both incidence of and complications after TKA, and (3) provided sufficient data for calculating the risk ratio (RR) or hazard ratio (HR) with a 95% confidential interval (CI). Exclusion criteria were as follows: (1) non-English articles, (2) studies without a non-smoking control group, (3) studies with incomplete data, and (4) study objective or intervention measures that failed to meet the inclusion criteria.

### Data extraction

Two authors selected the studies based on eligibility mentioned above criteria independently. If there was a disagreement between the two authors, it was settled by a mutual dialogue or involving a third author. The following data were extracted: (1) name of the first author, (2) year of publishment, (3) country of publication, (4) study design, (5) number of each group of patients, (5) gender ratio, (6) duration of follow-up, and (7) all the results.

### Quality assessment

The Newcastle-Ottawa Scale (NOS), a validated instrument for assessing the quality of observational research, was used to assess the quality of all included studies [19]. This scale was used to score the cohort studies by selection, comparability and outcome. Low, moderate, and high quality studies received scores of 0–3, 4–6, and 7–9, respectively. Only moderate and high quality studies were included in the analysis.

### Statistical analysis

Two authors entered the extracted data into Review Manager 5.4 (Cochrane Collaboration, Oxford, UK) independently. Some studies distinguished the smokers to former smokers and current smokers, but others combined them together. To unify the standard and study the long-term effects of tobacco on TKA, both current smokers and former smokers were considered as smokers. Dichotomous outcomes were expressed in terms of RR, and HR was used for the risk of TKA in some articles. Heterogeneity across investigations was evaluated using both the Chi-test and I^2^ statistics. Supposed the heterogeneity was considered statistically significant (*p* < 0.1 or I^2^ > 50%), the random-effects model was chosen to combine the data, while the fixed-effects model was adopted if no heterogeneity was found. When there was a high heterogeneity, sensitivity analysis was conducted, which means one research was removed at each turn, and the data from the remaining studies were pooled to explore potential reasons for high heterogeneity and assess the stability of the results. The publication bias test was not evaluated since no result was based on more than ten studies. A *p* < 0.05 was considered statistically significant.

## RESULTS

### Literature search

**FIGURE 1:**
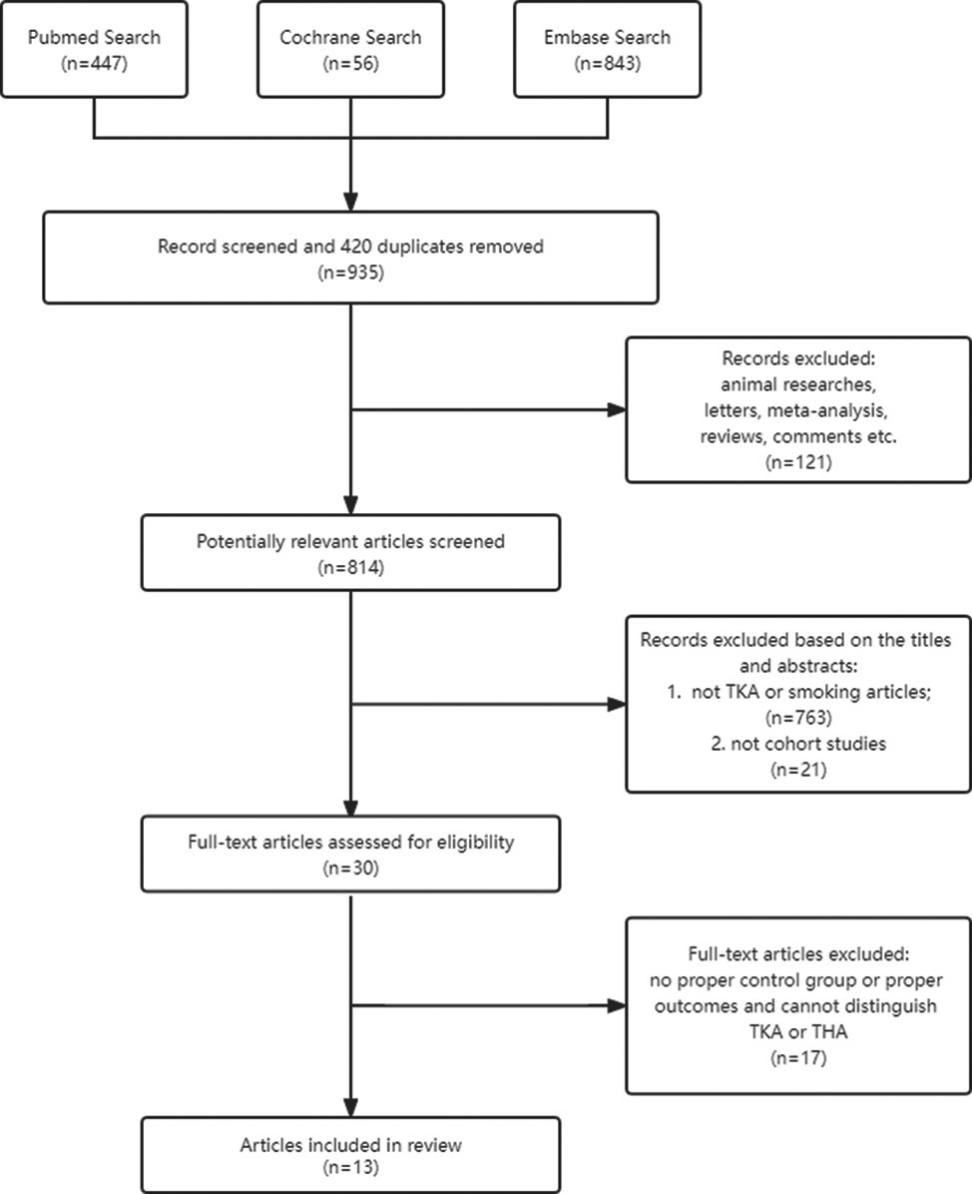
Flow chart of the assessment and selection of studies for the meta-analysis.

After combining searches from the three databases [PubMed (447), Cochrane (65), Embase (843)], we screened retrieved 1353 studies for review. There were 420 duplicates removed using an approach to ensure accuracy and prevent accidental loss of records. One hundred twenty-one articles on animal research, letters, meta-analyses, reviews, and comments were subsequently removed. The remaining 814 articles were screened by the title and abstract. Seven hundred sixty-three articles were excluded because they did not involve TKA or smoking, and 21 articles were excluded because they were not cohort studies. Out of 32 papers reviewed in detail, 17 were excluded due to no proper control group or proper outcomes (Supplement Table 2) [6-8,20-33] and 13 fit all the inclusion criteria and were used in our analysis. A flow chart showing the study assessment and selection process is presented in Figure 1.

### Study characteristics

These 13 studies, representing a total of 2 109 482 patients, came from Switzerland (n = 1), America (n = 4), Germany (n = 1), Spain (n = 2), UK (n = 2), Australia (n = 1), Norway (n = 1), and Singapore (n = 1). Six articles studied whether smoking affected the risk of TKA in patients diagnosed with OA or RA, and nine articles studied the effect of smoking on complications after TKA. The year of publication were from 2012 to 2020, the duration of follow-up ranged from 2 to 26 years, and the mean ages of the patients were between 45 and 74 years old. Eight studies utilized data from prospectively gathered cohorts and other seven studies were retrospective. The NOS score of all the studies was 6.54 ± 1.00, and all 13 studies had adequate quality for meta-analysis. All detailed study characteristics are summarized in Table 1 [12,14,34-43]

**TABLE 1:**
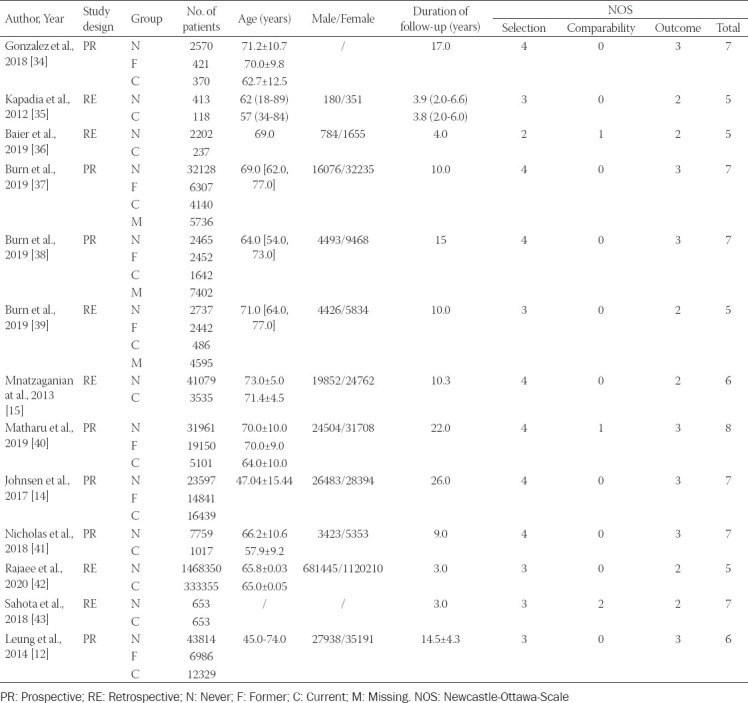
Demographic characteristics of included studies

### The incidence of TKA in diagnosed OA and RA patients

**FIGURE 2:**
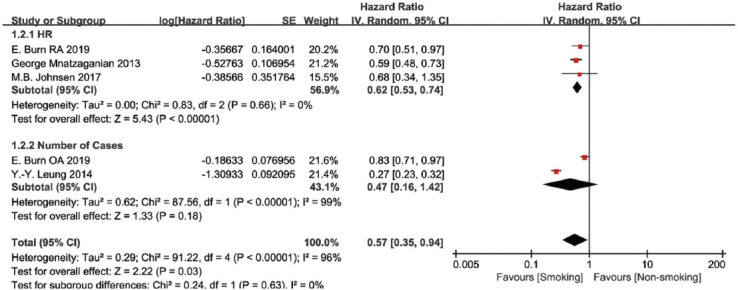
Forest plot of the association between smoking and the incidence of total knee arthroplasty in patients.

**SUPPLEMENT FIGURE 1:**
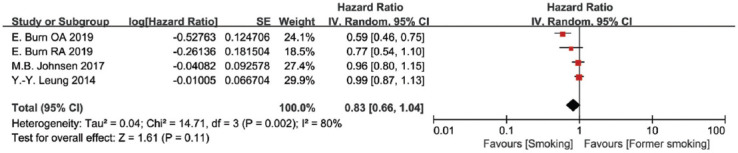
Forest plot of the association between smoking and the incidence of total knee arthroplasty in former smokers and current smokers

The incidence of TKA was mentioned in six studies with a total of 224,865 patients who were diagnosed with RA (n = 1) or OA (n = 4). There were two indicators to report the outcome: three studies used HR to report the outcome [14,15,38], and the other two studies reported the number of surgical cases [12,37]. According to the previous statistical methods [43,44], we approximately regarded the HR as similar to the RR and conducted the meta-analysis. As the heterogeneity between the studies and subgroups was significant (I^2^ > 50%), a random-effects model was employed in the meta-analysis. All the studies indicated that smoking can significantly reduce the incidence of TKA (summary RR = 0.57, 95% CI [0.35, 0.94], *p* < 0.01). To decline the high heterogeneity, subgroup analysis was performed according to different outcome indicators. Three studies that used HR had low heterogeneity (I^2^ = 0%), and meta-analysis of them indicated that smoking can decline the incidence of TKA (summary HR = 0.62, 95% CI [0.53, 0.74], *p* < 0.01). However, two studies using the number of surgical cases indicated that there was no statistical difference between smoking and incidence of TKA (summary RR = 0.47, 95% CI [0.16, 1.42], *p* = 0.18). Sensitivity analysis was also conducted, yielding similar results (Figure 2). Meanwhile, we analyzed the incidence of TKA in former smokers and current smokers, and found that there was no difference (summary HR = 0.83, 95% CI [0.66, 1.04], *p* = 0.11, Supplement Figure 1).

### Total complications

**FIGURE 3:**
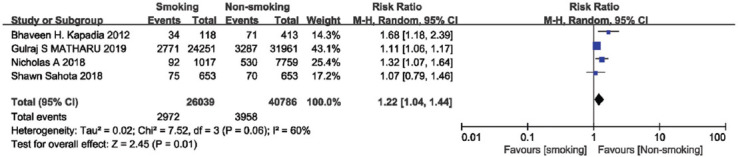
Forest plot of the association between smoking and total complications after total knee arthroplasty.

Four of the included studies have compared the total complications, including a total of 66 825 patients. Due to a significant heterogeneity between the studies (I^2^ = 60%), a random-effects model was used for the meta-analysis. The results showed that smoking can increase total complications after TKA (summary RR=1.22, 95% CI [1.04, 1.44], *p* = 0.01, Figure 3).

### Surgical complications

**FIGURE 4:**
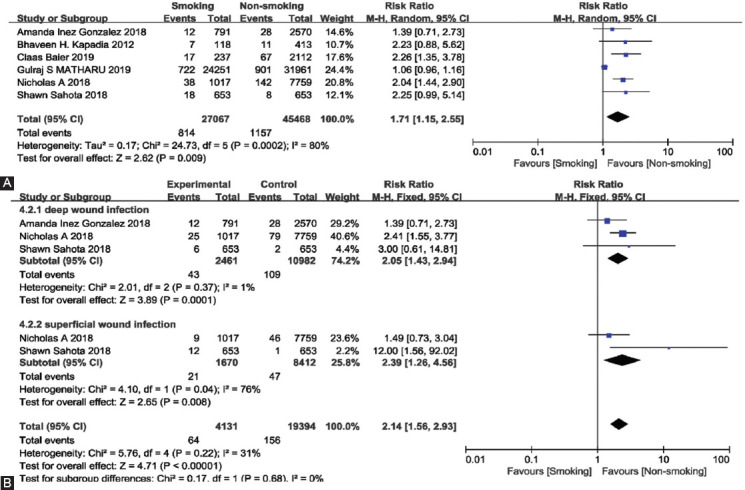
(A) Forest plot of the association between smoking and surgical complications after total knee arthroplasty, total surgical complications. (B) Forest plot of the association between smoking and surgical complications after total knee arthroplasty, deep wound infection, superficial wound infection, and wound dehiscence.

The surgical complications were analyzed in six studies with a total of 72 535 patients. A random-effects model was used because of significant heterogeneity (I^2^ = 80%). The results revealed that smoking can increase surgical complications after TKA (summary RR = 1.71, 95% CI [1.15, 2.55], *p* < 0.01, Figure 4A).

We divided the data on surgical complications into three subgroups for comparison between the two throngs to reduce the heterogeneity. The subgroups were deep wound infection and superficial wound infection. Three studies reported the specific classification of surgical complications, including 23,525 patients, with three studies involving deep wound infection, and two studies containing superficial wound infection. Since the heterogeneity between the studies and subgroups was not significant (I^2^ < 50%), a fixed-effects model was used for the meta-analysis. The results of the analysis revealed that deep wound infection (summary RR = 2.05, 95% CI [1.43, 2.94], *p* < 0.01) and superficial wound infection (summary RR = 2.39, 95% CI [1.26, 4.56], *p* < 0.01) were more present in the smoking group than in the non-smoking group (Figure 4B).

### Internal medicine complications

#### Pneumonia

**FIGURE 5:**
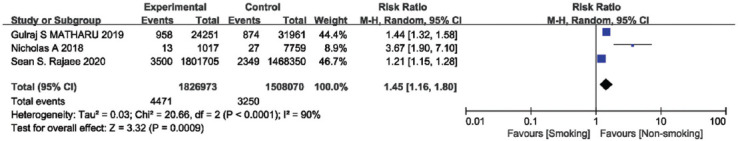
Forest plot of the association between smoking and pneumonia after total knee arthroplasty.

Three studies with a total of 3,335,043 patients were included, and the post-operative clinical outcome of pneumonia in the smoking and non-smoking groups was compared. A random-effects model was employed in the meta-analysis because the heterogeneity was significant (I^2^ > 50%). The result indicated that smoking increased the risk of pneumonia after TKA (summary RR = 1.45, 95% CI [1.16, 1.80], *p* < 0.01). Sensitivity analysis was also conducted, which produced similar results (Figure 5).

#### Blood transfusion

**FIGURE 6:**
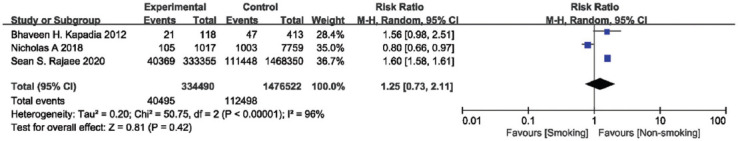
Forest plot of the association between smoking and blood transfusion after total knee arthroplasty.

In three studies involving a total of 1,811,012 patients, blood transfusion after TKA was compared. Since the heterogeneity between the studies was significant (I^2^ > 50%), a random-effects model was used for the meta-analysis. We found that there were no statistical differences between the two groups in terms of the blood transfusion (summary RR = 1.25, 95% CI [0.73, 2.11], *p* = 0.42, Figure 6). Sensitivity analysis detected that if Nicholas A’s study was excluded, heterogeneity would be greatly reduced (I^2^ = 0%) and the result would be different (*p* < 0.01).

#### Deep vein thrombosis

**FIGURE 7:**
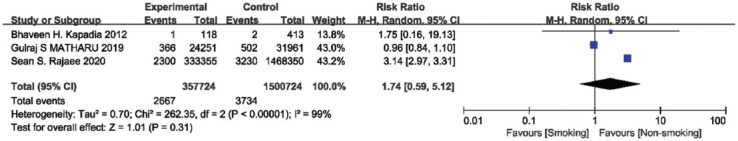
Forest plot of the association between smoking and deep vein thrombosis after total knee arthroplasty.

Three of the included studies compared deep vein thrombosis after TKA in the smoking and non-smoking groups with a total of 1,858,448 patients. A random-effects model was employed in the meta-analysis owing to significant heterogeneity (I^2^ > 50%). The results indicated that there were no statistical differences between the two groups concerning deep vein thrombosis (summary RR = 1.74, 95% CI [0.59, 5.12], *p* = 0.31, Figure 7). Sensitivity analysis showed that excluding Gulraj’s study, heterogeneity would be greatly reduced (I^2^ = 0%), and the result would be changed (*p* < 0.01).

#### Pulmonary embolism

**FIGURE 8:**
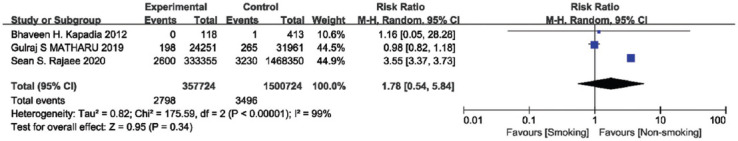
Forest plot of the association between smoking and pulmonary embolism after total knee arthroplasty.

Three studies, including a total of 1,858,448 patients, reported the number of patients who experienced pulmonary embolism after TKA. Since the heterogeneity was significant (I^2^ > 50%), a random-effects model was used for the meta-analysis. The results showed that there was no statistical difference between the two groups in terms of the pulmonary embolism (summary RR = 1.68, 95% CI [0.54, 5.84], *p* = 0.34, Figure 8). Sensitivity analysis elucidated that except for Gulraj’s analysis; there was a low heterogeneity (I^2^ = 0%) and the result would be statistically significant (*p* < 0.01).

#### Urinary tract infection

**FIGURE 9:**
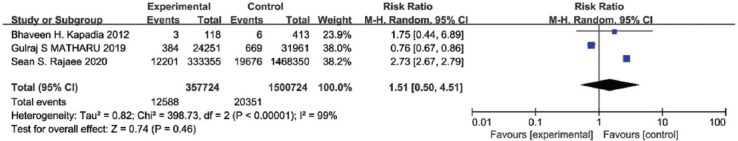
Forest plot of the association between smoking and urinary tract infection after total knee arthroplasty.

Urinary tract infection was mentioned in four studies, including a total of 1,858,448 patients. Due to the high heterogeneity (I^2^ > 50%), we used a random-effects model to analyze. Meta-analysis of the included studies suggested that no significant difference in the risk of implant dislocation was found between smokers and non-smokers (summary RR = 1.51, 95% CI [0.50, 4.51], *p* = 0.46, Figure 9). Sensitivity analysis showed that excluding Gulraj’s study, heterogeneity would be greatly reduced (I^2^ = 0%), and the result would be altered (*p* < 0.01).

### Revision

**FIGURE 10:**
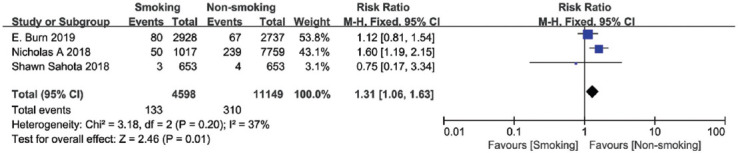
Forest plot of the association between smoking and revision after total knee arthroplasty.

In three studies involving a total of 15,747 patients, revision of the TKA by bacterial infection or removal of components for other causes were reported. There was no significant heterogeneity among these studies (I^2^ = 37%); hence, the fixed-effects model was employed in the meta-analysis. The results indicated a significantly increased risk of revision in the smokers after TKA (summary RR = 1.31, 95% CI [1.06, 1.63], *p* = 0.01, Figure 10).

### Mortality

**FIGURE 11:**
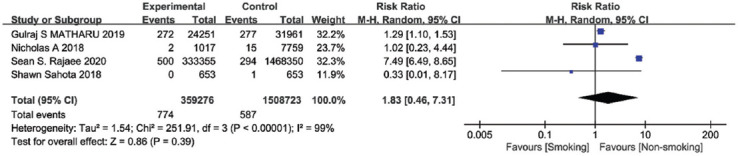
Forest plot of the association between smoking and mortality after total knee arthroplasty.

The mortality after TKA was reported in four studies with a total of 1,867,999 patients. Because of the strong heterogeneity of these experiments (I^2^ > 50%), a random-effects model was employed. Meta-analysis results indicated that there were no statistical differences between the two groups in mortality (summary RR=1.83, 95% CI [0.46, 7.31], *p* = 0.39, Figure 11). Sensitivity analysis revealed that excluding Sean’s study would significantly decrease heterogeneity (I^2^ = 0%), and the outcome would be altered (*p* < 0.01).

## DISCUSSION

This systematic review and meta-analysis sought to define the association between the use of tobacco and TKA better, including the impact before and after the operation. Six articles studied the risk of smoking for patients diagnosed with OA and RA who eventually needed to do TKA, which demonstrated that tobacco use for OA and RA patients decreased the risk of TKA by around 45%. Meanwhile, the results of this meta-analysis of the other nine studies indicated that tobacco use prior to TKA was associated with an increased risk of complications, especially surgical complications, including deep wound infection, superficial wound infection and wound dehiscence. These findings challenge the traditional view of smoking impact on TKA and deserve people’s attention.

TKA is the most effective treatment for patients with severe OA or RA which can relieve pain, but because of its invasive operation; it is generally considered to be the last choice for the treatment of OA or RA. In this study, five articles were used to evaluate the risk of TKA in OA or RA patients. Three articles used HR to report the outcome, and two articles used the number of surgical cases to report the outcome. Articles using HR came to a consistent conclusion: tobacco use reduced the risk of TKA. Moreover, these articles had low heterogeneity. However, the other two articles had a high heterogeneity, which may be due to the ethnic differences. These two articles found that there was no statistical difference between smoking and the risk of TKA. We roughly regarded HR as RR using the previous statistical methods [44,45] and summarized and analyzed all five papers. The results showed that smoking could significantly reduce the risk of TKA.

This result seems to differ from our traditional view, but there are two possible explanations. The first one is that body mass index (BMI) is generally lower in smokers, and the second is that nicotine has a protective effect on chondrocytes. Obesity is a recognized risk factor for knee OA [46], as well as a significant confounding factor in the smoking-OA relation, as smokers are usually thinner than non-smokers. Zeng et al. [47] found smoking cessation was linked to a higher risk of TKA in people with knee OA, and this link was found to be attributable to the weight gain after quitting smoking. Hence, non-smokers are prone to having a higher BMI compared with smokers, which makes non-smokers more likely to have joint replacements. However, to avoid the impact of BMI, Leung et al. [12] found the existence of a statistically significant inverse relationship between smoking and TKA risk after adjusting for BMI, as well as among people with a BMI of less than 25 kg/m^2^, indicating that the inverse relationship between smoking and a severe knee OA is independent of obesity. Further cell experiments elucidated that nicotine in cigarettes plays a key role as a protective agent for OA [48]. Nicotine stimulated the proliferation of articular chondrocytes isolated from the knee joints of both the healthy and OA patients in a concentration-dependent manner. It can also upregulate collagen production in chondrocytes isolated from the healthy humans femoral head [48]. In both forms of chondrocytes, nicotine also increased the expression of cartilage-specific type II collagen.

Post-operative complications are a problem that will never be eliminated entirely. All surgeons want to reduce the incidence of post-operative complications, but some are inevitable. In terms of the impact of smoking on TKA post-operative complications, the results of this study are similar to those of previous studies [16], indicating that smoking can increase TKA post-operative complications. This systematic review and meta-analysis mainly researched the total complications, surgical complications (including deep wound infection, superficial wound infection and wound dehiscence) and internal medicine complications (including pneumonia, blood transfusion, deep vein thrombosis, pulmonary embolism, and urinary tract infection). Five articles analyzed the relationship between smoking and total complications and concluded that smoking increases the risk of total complications. Although these articles had moderate heterogeneity, the result was stable by sensitivity analysis.

Further subdividing the total complications, we found that surgical complications mainly caused them. Smokers are more likely to develop deep wound infection, superficial wound infection and wound dehiscence after surgery in comparison to non-smokers by 1.07-fold, 1.08-fold, and 2.83-fold, respectively. Sensitivity analysis confirmed the stability of the positive association between smoking and the risk of surgical complications. *In vivo* experiment, a custom-made chamber with airflow for rats to inhale cigarette smoke continuously, was established by Chang et al., and it was found that cigarette smoke inhibited early angiogenesis using a femoral osteotomy model, analyzed by radiograph and micro-CT imaging and various biomechanical and biological tests [49]. Other experiments have also demonstrated that tobacco accelerated the pronounced initial inflammatory response [50], suppressed the immune system [51] and increased infection incidence.

Regarding internal medicine complications, pneumonia was the only statistically significant complication, which means smoking has little effect on post-operative internal medical complications except for pneumonia. Nevertheless, all the internal medicine complications had a high heterogeneity, and their results were unstable using sensitivity analysis. The reason may be that the number of literatures included in the study was too small, and the difference in sample size between studies was too large. For example, Sean et al. [42] included 1,801,705 patients in their study, while Shawn et al. [43] had only 1306 patients, a difference of more than 1300 times. Such a significant difference in sample size may be one of the reasons for the considerable heterogeneity. This problem is also raised when calculating mortality. More research is needed to better understand the relationship between smoking and medical problems and mortality following TKA.

Revision is another indicator that surgeons are very concerned about after surgery. Common reasons for post-operative revision include instability, implant loosening, arthrofibrosis, and deep infections [35]. Analyzing 18 years of data from Hospital Episode Statistics Admitted Patient Care, Burn et al. [39] found smoking did not appear to have a meaningful impact on the risk of revision following TKA. However, through the combined analysis of the data from the four articles, smokers were 1.84 times more likely to have a revision after TKA than non-smokers. According to Bedard et al. [41] analysis, smokers were at a higher risk of infection and wound complications, resulting in a high rate of revision, which was the same as the results of our study.

Smoking cessation is another hot spot that orthopedists focus on. In this study, seven included studies distinguished the former smokers and current smokers, four of them focused on the incidence of TKA [12,14,37,38], one focused on the prosthetic joint infection [34], one focused on the complications [15], and one focused on revision [39]. After analyzing the four studies, there was no difference between former smokers and current smokers, indicating that smoking cessation cannot change the existing effects of tobacco on the knee joint. For the other studies, revision had no difference, but the rate of prosthetic joint infection and post-operative complications decreased in the former smokers compared to current smokers. This is similar to some studies focused on smoking cessation [52-54]. Smoking cessation has little effect on the incidence of TKA, but decreases post-operative problems in patients; thus, it should be encouraged once they are diagnosed with knee disease.

Although these data revealed a very interesting impact of smoking on TKA, our conclusion must be treated with caution and considered in the presence of possible limitations. Firstly, the meta-analysis included just cohort studies, and observational studies can only provide details about correlations, but not the causality. Secondly, the number of included studies was insufficient, especially in medical complications and mortality indicators. This lead up to the excessive heterogeneity of the two indicators, and the results were unstable. Moreover, the definitions of smoking status were diverse and ambiguous. Most studies judged patients’ smoking status only based on their memories; therefore, the amount of smoking cannot be quantified. This would cause the subjective deviation of the patients, which may affect the results.

Meanwhile, despite the fact that these studies attempted to match smokers’ and non-smokers’ other conditions, we cannot rule out the possibility that smoking patients have more surgical contraindications, resulting in lower surgery rates. Patients’ symptoms or radiological changes are better ways to evaluate the progress on knee disease, but the current researches have different opinions. Felson et al. insist that smoking modestly protects against the development of knee OA by assessing weight-bearing knee radiographs [55,56]. Wilder et al. also use serial radiographs to evaluate the progress of knee disease, but they think there is no association between smoking and knee disease [57]. Analyzing combine magnetic resonance imaging and Visual Analogue Scale pain score, Amin et al. conclude that smokers sustain greater cartilage loss and have more severe knee pain in OA than non-smokers [58]. We still need more clinical and basic medical experiments to explore deeply.

## CONCLUSION

In conclusion, this study suggests that tobacco use can enhance the incidence of complications after TKA for both OA and RA patients, especially when it comes to surgical complications and pneumonia, but decreases the risk of TKA implantation in the population. Although smoking reduces the risk of TKA, we would not recommend smoking to prevent the occurrence or progression of OA or RA. However, smoking cessation should be encouraged, as it cannot affect the existing protective effect of tobacco on the knee joint, and can reduce post-operative complications. The deeper meaning is also that chemoprophylaxis drugs based on nicotine analogs should be further studied with the aim of producing medicine that can delay severe knee OA or RA. The possibility of patient selection due to comorbidity attributed to smoking must be ruled out in further studies.

## Figures and Tables

**SUPPLEMENT TABLE 1:**
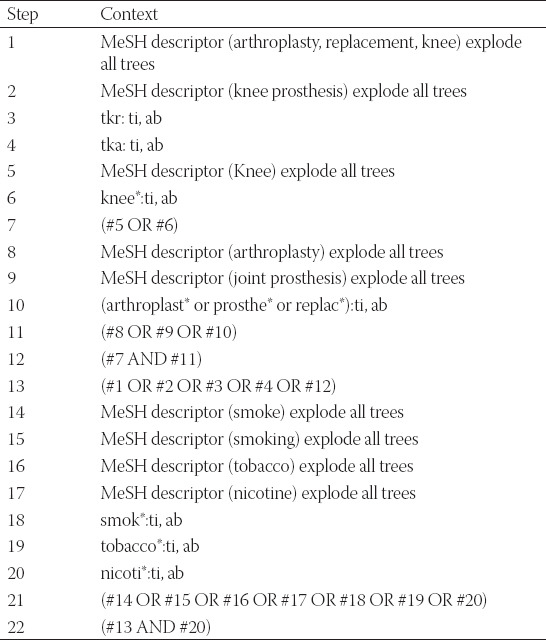
Research strategy

**SUPPLEMENT TABLE 2:**
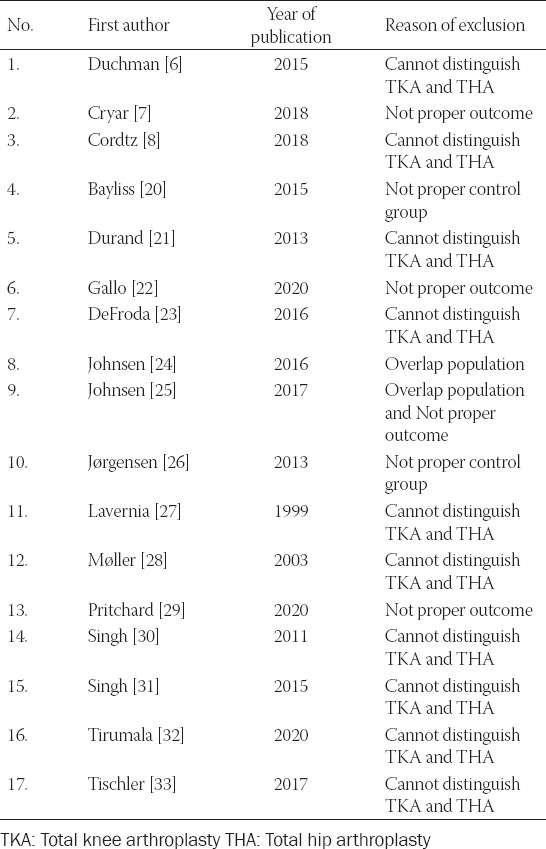
Reason of exclusion after a full-text reading
